# Synthesis and Evaluation of Thermoresponsive Renewable Lipid-Based Block Copolymers for Drug Delivery

**DOI:** 10.3390/polym14173436

**Published:** 2022-08-23

**Authors:** Huiqi Wang, Aman Ullah

**Affiliations:** Department of Agricultural, Food and Nutritional Science, University of Alberta, Edmonton, AB T6G 2P5, Canada

**Keywords:** fatty acid, PNIPAM, block copolymer, microwave-assisted, self-assembly, drug delivery

## Abstract

Polymeric micelle forming from self-assembly of amphiphilic macromolecules is one of the most potent drug delivery systems. Fatty acids, naturally occurring hydrophobic lipid components, can be considered as potential candidates for the fabrication of block copolymer micelles. However, examples of synthesis of responsive block copolymers using renewable fatty acids are scarce. Herein, we report the synthesis, characterization and testing of block copolymer micelles composed of a renewable fatty-acid-based hydrophobic block and thermoresponsive hydrophilic block for controlled drug delivery. The block copolymers of functionalized fatty acid and poly(N-isopropylacrylamide) (PNIPAM) were prepared via consecutive microwave-assisted reversible addition fragmentation chain transfer (RAFT) polymerization. The block copolymers with variable hydrophobic block length self-assembled in aqueous media and formed spherical nanoparticles of ~30 nm with low critical micelle concentration (CMC). To demonstrate the proof-of-concept, carbamazepine (CBZ) was used as a hydrophobic model drug to evaluate the performance of these micelles as nanocarriers. The in vitro drug release tests were carried out below (25 °C) and above (37 °C) the lower critical solution temperature (LCST) of the block copolymer. The drug release showed obvious temperature-triggered response and an accelerated drug release at 37 °C.

## 1. Introduction

Many drug molecules that are currently on the market suffer from short half-life, in vivo instability, poor bioavailability and rapid degradation. Since the 1960s, various drug delivery systems, typically polymers including polymeric micelles, liposomes, dendrimers and nanotubes have been developed [1,2,3,4,5,6]. Their ability to transport therapeutic agents in a targeted and controllable manner is the major goal of modern medicine. Polymeric micelles are nanosized structures formed by self-assembly of amphiphilic block copolymers in aqueous solution. They have two distinct regions with a hydrophilic shell and hydrophobic core that facilitates the solubilization of poorly soluble pharmaceuticals. There are many advantages of using micelles as drug delivery vehicles over others, such as their ease of preparation, relatively high stability and size advantages enabling passive targeting of tumors through enhanced permeability and retention (EPR) effect [7,8,9]. Additionally, micelles with versatile functions and characteristics can be easily designed by altering the type and chemical structure of block copolymers. To date, several polymeric micelles as drug delivery vehicles have been authorized or are in clinical trials [10,11]. 

To achieve site-specific drug targeting and on-demand release, research efforts have been devoted to develop advanced drug delivery systems [12,13]. One such example is smart delivery systems, which involve the utilization of stimuli-responsive polymers. These carriers can hold drugs in the course of transport and only release them in response to internal or external stimuli, e.g., heat, pH, enzyme, light and ultrasound, due to a sharp change in their physical properties [14,15,16,17,18]. Temperature is one of the most extensively investigated stimuli for drug delivery. The drug release from thermoresponsive carriers can be either internally observed in certain pathological tissues (e.g., ~42 °C at tumor sites) [19] or externally achieved by applying an external heating source. Thermoresponsive polymers exhibit a hydrophilic nature below a certain temperature, known as lower critical solution temperature (LCST), but become hydrophobic and collapse as temperature increase above their LCST. The LCST values within a desired range can be modulated by introduction of the hydrophobic or hydrophilic co-monomers [20,21]. Poly(N-isopropylacrylamide) (PNIPAM) is the most well-studied thermoresponsive polymer with an LCST around 32 °C in aqueous media, which is close to the physiological temperature of the human body. PNIPAM and its copolymers have been extensively exploited for various biomedical applications [22,23,24]. There are also many examples of PNIPAM-based amphiphilic block copolymers for drug delivery applications, where the hydrophobic segments are comprised of polystyrene [25], poly(methyl methacrylate) [26], poly(ε-caprolactone) [27], etc. 

Synthesis of block copolymers incorporating biodegradable and biocompatible monomers are highly attractive for micelle preparation due to their potential sustained drug release and low toxicity [28,29]. In recent years, the use of monomers from renewable feedstocks to replace fossil-based monomers with an aim to promote greener solutions has received special attention. In this regard, fatty acid moieties from renewable plant oils can be considered as great candidates and alternatives to some synthetic hydrophobic monomers due to their good biocompatibility, biodegradability, easy availability and proper hydrophobicity. Up to now, most studies have focused on the application of fatty-acid-based polymers into biocomposites, thermoplastics and elastomers [30,31,32]. While there are a few examples of fatty-acid-based block copolymers using atom transfer radical polymerization (ATRP) or reversible addition-fragmentation chain transfer (RAFT) [33,34,35,36,37], few attempts have been made in terms of their applications as amphiphilic block copolymers for drug delivery. To the best of our knowledge, the microwave-assisted synthesis of thermoresponsive block copolymers from fatty acids and NIPAM and their controlled delivery has not been reported. 

**Scheme 1:**
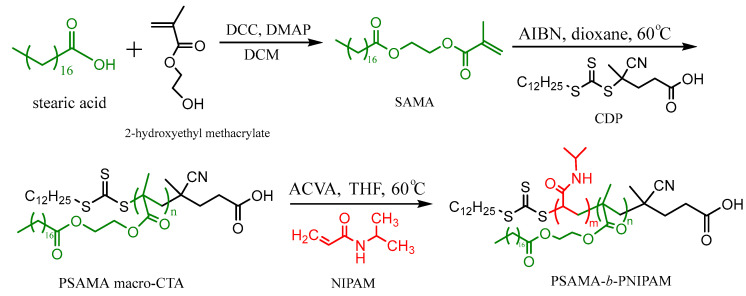
Synthetic route for amphiphilic block copolymer PSAMA-*b*-PNIPAM via microwave-assisted RAFT polymerization.

In this study, we report the synthesis and characterization of an amphiphilic block copolymer containing fatty-acid-derived polymer poly(2-methacryloyloxy) ethyl stearate (PSAMA) and thermoresponsive polymer PNIPAM through RAFT polymerization under microwave irradiations (Scheme 1). PSAMA-*b*-PNIPAM block copolymers with narrow polydispersity index (PDI) and well-controlled lengths of different block ratios were prepared and self-assembled in aqueous medium via the combination of co-solvent evaporation and dialysis method. Afterwards, the effect of balance between hydrophilic-hydrophobic interactions on particle size, morphology and critical micelle concentration (CMC) of the polymeric micelles was investigated. Carbamazepine (CBZ), an antiepileptic and anticonvulsant drug, was selected as a hydrophobic model drug to evaluate the drug encapsulation ability of PSAMA-*b*-PNIPAM micelles. Moreover, the in vitro thermoresponsive drug release behavior of PSAMA-*b*-PNIPAM micelles was also studied to further explore their potential application as drug carriers.

## 2. Materials and Methods

### 2.1. Materials

N-isopropylacrylamide (NIPAM, >99%, Sigma, St. Louis, MO, USA) was recrystallized from n-hexane before use. Furthermore, 2, 2’-Azobisisobutyronitrile (AIBN, 98%) and 4,4’-Azobis(4-cyanovaleric acid) (ACVA, ≥98%) were obtained from Sigma (St. Louis, MO, USA) and recrystallized from methanol prior to use. Stearic acid (>98%) was purchased from TCI America (Tokyo, Japan) and used as received. Additionally, 4-Cyano-4-[(dodecylsulfanylthiocarbonyl)sulfanyl]pentanoic acid (CDP, 97%), dicyclohexylcarbodiimide (DCC, 99%), 4-dimethylaminopyridine (DMAP, 99%), 2-hydroxyethyl methacrylate (HEMA, ≥99%) carbamazepine (CBZ, ≥98%), 1,3,5-trioxane (≥99%), pyrene (98%) and sodium sulphate anhydrous (Na_2_SO_4_, 99%) were purchased from Sigma (St. Louis, MO, USA) and used without any further purification. Then 1,4-Dioxane (99%, Fisher Scientific, Waltham, MA, USA) and tetrahydrofuran (THF, ≥99%, Sigma, St. Louis, MO, USA) was purified by being passed through a short alumina column before use. Chloroform-d (99.8 atom %D), dichloromethane (DCM, ≥99.5%) and ethyl acetate (EtOAc, ≥99.5%) were obtained from Sigma (St. Louis, MO, USA), while sodium bicarbonate (NaHCO_3_, 99.8%) and sodium chloride (NaCl, 99.9%), diethyl ether (≥99%), methanol (99.8%) and hexane (98.5%) were purchased from Fisher Scientific (Waltham, MA, USA).

### 2.2. Synthesis of 2-(Methacryloyloxy) Ethyl Stearate (SAMA)

Fatty-acid-based monomer SAMA was synthesized according to the previous reported method [35,38] with slight modification. Typically, stearic acid (52.7 mmol, 15 g) and DMAP (5.27 mmol, 0.6 g) were first dissolved in dry 50 mL DCM and kept in an ice-water bath. The mixture was purged with nitrogen gas while stirring. After 15 min, a solution of DCC (58.0 mmol, 12.0 g) in a minimum volume of DCM was added. HEMA (58.0 mmol, 7.5 g) was subsequently added drop by drop over a period of 20 min. The ice-water bath was then removed and the reaction mixture stirred for another 24 h at room temperature. After the reaction, the mixture was filtered to remove extra reagents. The obtained filtrate was washed with distilled water, saturated NaHCO_3_ solution and brine solution in sequence and dried over anhydrous Na_2_SO_4_. The solvent was removed by rotary evaporator and purified using a silica gel column with hexane/EtOAc (95: 5, *v/v*) as the eluent. The final product with 86% yield (12.9 g) was obtained and its structure was confirmed by ^1^H NMR and FTIR. 

^1^H NMR (400 MHz, CDCl_3_): δ (ppm) = 6.11 and 5.58 (C=CH_2_, 2H, d), 4.32 (OCH_2_CH_2_O, 4H, t), 2.31 (O=CCH_2_, 2H, t), 1.94 (CH_2_=CCH_3_, 3H, s), 1.61 (O=CCH_2_CH_2_, 2H, m), 1.27 ((−CH_2_)n, 28H, m) and 0.87 (–CH_2_CH_3_, 3H, t).

### 2.3. Synthesis of Homopolymers

PSAMA and PNIPAM was synthesized using 4-Cyano-4-[(dodecylsulfanylthiocarbonyl)sulfanyl]pentanoic acid (CDP) as the chain transfer agent (CTA) at 60 °C. An example of polymerization of SAMA is described as follows: SAMA monomer (1309 mg, 3.3 mmol), CTA (53.3 mg, 0.13 mol), AIBN (4.3 mg, 0.0264 mmol) and trioxane (80 mg, 0.89 mmol) (internal standard for monomer conversion calculation) were added into a dry glass vial and dissolved in 2.4 mL 1,4-dioxane. The mixture was stirred and purged with nitrogen for 20 min. After that, the vial was placed in the microwave reactor and the reaction was conducted under programmed conditions at 60 °C for a certain time. The reaction was stopped by cooling to room temperature with an ice-water bath and exposing to air. The homopolymer PSAMA was purified by precipitation from methanol at least three times and dried in a fuming hood at room temperature. In the case of polymerization of NIPAM, the precipitation step was carried out with diethyl ether.

### 2.4. Synthesis of PSAMA-b-PNIPAM Block Copolymer 

The resulting homopolymers were used as macro-CTA for the synthesis of block copolymer. For this, a typical example when PSAMA was used as the macro-CTA has been described as follows. Into a 10 mL glass vial equipped with a stirrer bar, NIPAM (484.3 mg, 4.28 mmol), PSAMA macro-CTA (67.5 mg, 0.014 mmol), ACVA (0.78 mg, 0.003 mmol), trioxane (40 mg, 0.45 mmol) and 1.6 mL of THF were added. After mixing and deoxygenating with dry nitrogen for 20 min, the reaction was conducted in the microwave for 25 min at 60 °C. The reaction was quenched by cooling the polymer to room temperature and exposing to air. To obtain purified PSAMA-*b*-PNIPAM, the synthesized polymer was precipitated twice in cold hexane followed by twice in diethyl ether. The final product was dried in a fume hood at ambient temperature prior to characterization with GPC, ^1^H NMR and FTIR.

### 2.5. Characterization and Instruments

Molecular weight and PDI of polymers were determined by gel permeation chromatography (GPC). The GPC instrument consisted of an Agilent 1200 series pump and autosampler, Agilent 1200 series evaporative light scattering detector and one Phenogel 5 µm 500A column (300 × 4.6 mm^2^), using THF as the eluent at a flow rate of 0.5 mL min⁻^1^. Samples were prepared at a concentration of 0.5 mg mL⁻^1^. A series of polystyrene standards were used for calibration of the instrument. ^1^H NMR spectra were recorded on a Varian INOVA spectrometer operating at 399.79 MHz at 27 °C. All samples were dissolved in deuterated chloroform for the measurements. Purified homopolymers and block copolymers were dried completely and mixed with KBr powder to prepare KBr pellets. FTIR analysis was conducted on IRSprit-L FTIR spectrophotometer (Shimadzu, Japan) with the spectral scanning scope of 400–4000 cm⁻^1^, the number of scans was 40 and resolution was 4 cm⁻^1^. Particle size in aqueous solution was measured by dynamic light scattering (DLS) using a Malvern Zetasizer Nano-ZS instrument equipped with a 4.0 mW He-Ne laser operating at a wavelength of 633 nm and scattering angle of 173°. All samples were prepared at a concentration of 0.25 mg mL⁻^1^ and measured in triplicate at 25 °C. Fluorescence excitation spectra and UV-vis measurement were performed on a SpectraMax M3 Multi-mode Microplate Reader. TEM analysis was conducted on an FEI Morgagni 268 (Hillsboro, OR,, USA) equipped with a Gatan Orius CCD camera, operating at an acceleration voltage of 80 kV. A drop of polymeric micelles suspension was deposited onto a carbon-coated copper grid and left for 5 min under ambient conditions. Excess solution was wicked away with filter paper. All samples were negatively stained with phosphotungstic acid (PTA) to improve contrast on the images. A concentration of 0.25 mg mL⁻^1^ was used for the analysis.

### 2.6. Determination of Critical Micelle Concentration (CMC)

The CMC of the block copolymers PSAMA-*b*-PNIPAM was determined by fluorescence measurements using pyrene as a probe. Briefly, 20 µL of pyrene solution with 3 × 10⁻^4^ M concentration in acetone was added into a series of vials and then the acetone was allowed to evaporate. Different concentrations of polymer solution ranging from 5 × 10⁻^4^ to 0.2 mg/mL were prepared and added into the vials while the final concentration of pyrene in each vial was maintained at 6 × 10^−7^ M. The solutions were kept at room temperature for 24 h before measurements to equilibrate pyrene and micelles. The excitation spectra were recorded from 300 to 350 nm with a fixed emission wavelength of 390 nm. The ratio of fluorescence intensity at two wavelengths, 337 and 333 nm, in the excitation spectra was plotted against the logarithm of the block copolymer concentration [39]. 

### 2.7. Micelle Formation

The micelles of the block copolymer were prepared using the combination of co-solvent evaporation and dialysis method. Briefly, 10 mg of PSAMA-*b*-PNIPAM was dissolved in 5 mL THF and equal amount of distilled water was then added drop-wise (ca. 1 droplet per 10 s) to the glass vial under gentle stirring. The prepared solution in the open vial was stirred continuously for 6 h and transferred into dialysis tubing (MW CO = 3.5 KDa). The solution was dialyzed against distilled water overnight to completely remove THF and then lyophilized.

### 2.8. Drug Loading within Micelles

Carbamazepine (CBZ) was loaded into PSAMA-*b*-PNIPAM micelles using the same method as described above, where 1 mg of CBZ and 10 mg of PSAMA-*b*-PNIPAM were dissolved in THF in the first step. To calculate drug loading efficiency (DLE) and drug loaded content (DLC), the freeze-dried CBZ-loaded sample was dissolved in THF and the amount of encapsulated drug in polymeric micelles was analyzed by UV-visible spectrophotometer at 287 nm. A linear standard curve between CBZ concentration and UV absorbance at 287 nm was established in advance. The DLE and DLC were calculated according to the following formula [40,41]:

(1) $$\mathrm{DLE}\;(\%)=\;\frac{\mathrm{Mass}\;\mathrm{of}\;\mathrm{loaded}\;\mathrm{drug}\;}{\mathrm{Mass}\;\mathrm{of}\;\mathrm{drug}\;\mathrm{in}\;\mathrm{feed}}\times \;100$$

(2) $$\mathrm{DLC}\;(\%)=\frac{\mathrm{Mass}\;\mathrm{of}\;\mathrm{loaded}\;\mathrm{drug}}{\mathrm{Mass}\;\mathrm{of}\;\mathrm{drug}-\mathrm{loaded}\;\mathrm{micelle}\;}\;\times 100$$

### 2.9. In Vitro Drug Release Study

Drug release behavior of CBZ-loaded PSAMA-*b*-PNIPAM micelles in vitro was studied via the dialysis method. A solution of CBZ-loaded polymeric micelles (1 mg/mL) was placed into a dialysis membrane (MW CO = 100–500 Da). This dialysis tube was immersed in a 35 mL physiological buffer (0.01M PBS, pH 7.4) and incubated at 37 or 25 °C under gentle stirring. At scheduled time intervals, 2 mL of samples were taken out for measurement and an equal volume of fresh PBS solution was refilled to maintain the sink conditions. The release amount of CBZ was analyzed and monitored by UV-visible spectrophotometer. 

## 3. Results and Discussion

### 3.1. Synthesis of Block Copolymer PSAMA-b-PNIPAM

As one of the most versatile controlled radical polymerization (CRP) techniques, RAFT polymerization has been considerably employed for fabrication of block copolymers [42,43]. It provides a convenient route to prepare (co)polymers with predicted compositions, topologies and functionalities [44,45]. As depicted in Scheme 1, block copolymers of PSAMA and PNIPAM were synthesized by sequential monomer addition in two steps. To begin with, homopolymers of PSAMA or PNIPAM using CDP as the RAFT agent were synthesized. The selection of an appropriate thiocarbonylthio moiety of the RAFT agent is the key to successful controlled polymerizations [46]. The CDP was chosen as RAFT agent due to its ability to control polymerization of both SAMA and NIPAM monomers [35,47]. Polymerizations were carried out in 1,4-dioxane at 60 °C and the results are presented in Table 1. Both PSAMA and PNIPAM were obtained with different molecular weights and narrow molecular weight distributions.

The sequence of monomer addition has a great effect on the product of RAFT polymerization for the synthesis of block copolymers. In this study, both PSAMA and PNIPAM were employed as the macro-CTA to further copolymerize with the second monomer to produce the block copolymer. However, it was observed that the block copolymer could only be formed when PSAMA was used as the first block (macro-CTA). When starting with a PNIPAM macro-CTA, the final products were found to be the mixture of PSAMA homopolymer and PNIPAM homopolymer. Considering the RAFT mechanism for block copolymerization, the homopolymer (macro-CTA) with thiocarbonylthio group should have a higher transfer ability and better leaving ability to allow the growth of the second monomer [48,49,50] which was opposite when PNIPAM was used as a macro-CTA, therefore block-copolymerization on NIPAM macro-CTA was not successful. Consequently, PSAMA was used as the macro-CTA and PSAMA-*b*-PNIPAM block copolymers with narrow PDI and well-controlled length of different blocks were synthesized. The results are summarized in Table 1.

The polymerization reactions were performed under microwave irradiation and conventional heating was also employed for the purpose of comparison. Table 1 shows that when producing PSAMA homopolymers, compared to oil bath heating (300 min), microwave irradiation (20 min) greatly shortened the reaction time, which also provides a rapid and efficient way for synthesis of block polymers using lipid-based monomers and NIPAM. The block copolymers were prepared within 25 min while it usually takes 12–48 h to produce block copolymers with conventional oil bath heating [42].

### 3.2. Characterization of PSAMA-b-PNIPAM

**Figure 1:**
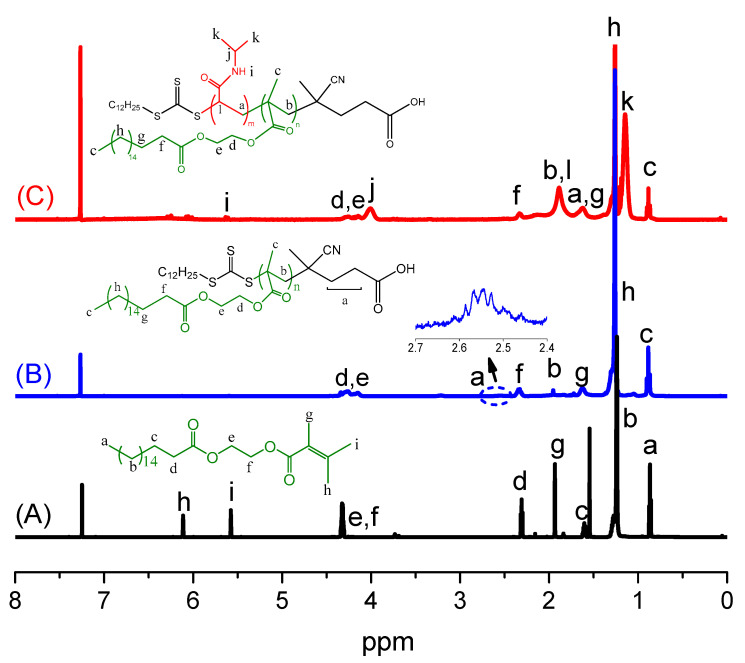
^1^H NMR spectrum of (**A**) SAMA, (**B**) PSAMA and (**C**) PSAMA-*b*-PNIPAM.

The chemical structures of the synthesized monomer SAMA, homopolymer PSAMA and block copolymer PSAMA-*b*-PNIPAM were characterized by ^1^H NMR spectroscopy. The assignations of various proton signals marked with different letters are displayed in Figure 1. In the ^1^H NMR spectrum of monomer SAMA, the proton peaks labeled as *h* and *i* were attributed to vinyl proton signals. Monomer conversion was calculated by comparing the integration of these vinyl protons before and after polymerization using 1,3,5-trioxane as an internal standard (δ = 5.10 ppm). The signals at 2.39–2.62 ppm found in the spectrum of the PSAMA homopolymer corresponded to the HOOC–CH_2_–CH_2_–C(CN)(CH_3_)– moiety in the RAFT agent [51]. We determined the degree of polymerization (DP) of PSAMA macro-CTA by end-group analysis, based on the integral ratio of the repeating chain protons at 4.12–4.37 ppm, derived from the side chain –O–CH_2_–CH_2_–O–, to methylene protons H_a_ in the CTA agent (at 2.39–2.62 ppm). The M_n,NMR_ of PSAMA was calculated from DP values in combination with the molecular weights of the RAFT agent by using the equation: M_n,NMR_ = DP_n,PSAMA_ × molecular weight of SAMA + molecular weight of CTA. The NMR spectrum of PSAMA-*b*-PNIPAM proved the presence of all characteristic peaks from each block. In order to determine the molar ratio of PSAMA to PNIPAM in the block copolymers, the integrated peak area at 4.12–4.37 ppm from PSAMA segment was compared with that of methine proton (*H_j_* at 3.88–4.10 ppm) from PNIPAM polymer backbone. The *M_n_* value of block copolymer measured by NMR technique was calculated as: M_n,NMR_ = DP_n, PNIPAM_ × molecular weight of NIPAM + DP_n, PSAMA_ × molecular weight of SAMA + molecular weight of CTA.

**Figure 2:**
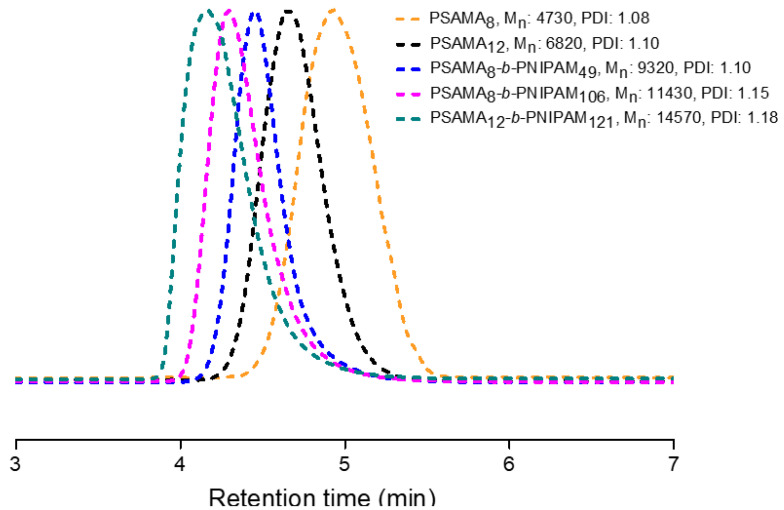
GPC traces of block copolymer PSAMA-*b*-PNIPAM and its PSAMA macro-CTA.

Starting with PSAMA_8_ and PSAMA_12_ as macro-CTA, three PSAMA-*b*-PNIPAM block copolymers with variable block lengths using different molar ratios of monomer to macro-CTA were synthesized, at a fixed ratio of (macro-CTA)/(AIBN) = 0.2. The GPC spectra of homopolymers and block copolymers are shown in Figure 2. GPC curves of PSAMA-*b*-PNIPAM moved towards the higher molecular weight region in contrast to PSAMA macro-CTA while maintaining low PDI (<1.2). The GPC peaks were all unimodal and symmetric without any bimolecular termination products or unreacted macro-CTA. Therefore, GPC results further confirmed the successful chain extension of PSAMA macro-CTA and block copolymerization. The molecular weights measured by GPC matched reasonably well with M_n,NMR_ based on DP, in spite of there being some discrepancy with the theoretical molecular weights calculated on the basis of monomer conversion by NMR. Note that the molecular weights measured by GPC are relative values with respect to polystyrene standards.

**Figure 3:**
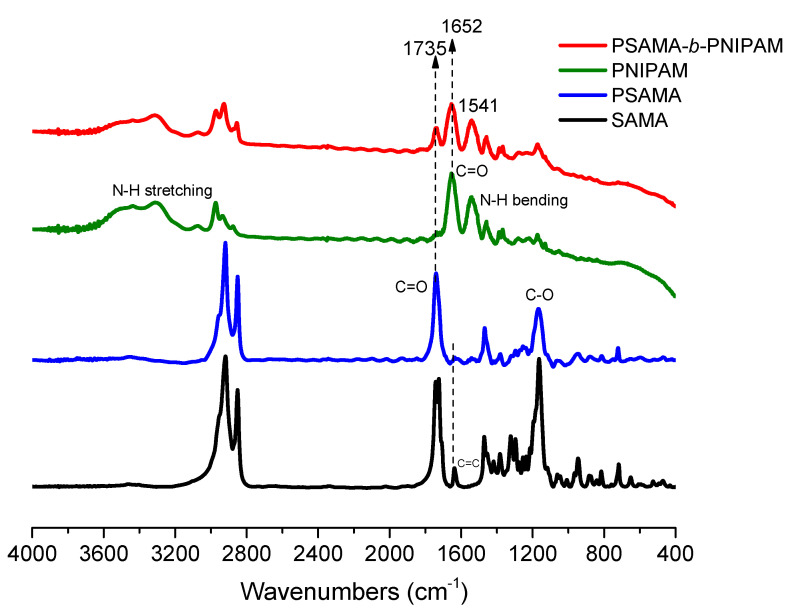
FTIR spectrum of SAMA, PSAMA, PNIPAM and PSAMA-*b*-PNIPAM.

The structural differences of monomer, homopolymers and block copolymer were also studied by FTIR. As illustrated in Figure 3, in the IR spectrum of the SAMA monomer, the peak for C=C was observed at 1645 cm⁻^1^. The peaks at 1724 and 1742 cm⁻^1^ corresponded to the two ester carbonyl groups’ stretching. It was found that after polymerization, C=C double-bond peak disappeared and there was a broad C=O peak at 1735 cm⁻^1^ in the spectrum of PSAMA. In the spectrum of PSAMA-*b*-PNIPAM, all characteristic absorption peaks can be observed associated with both blocks. For example, it includes stretching vibration of ester carbonyl group in PSAMA at 1735 cm⁻^1^ and secondary amide C=O stretching at 1652 cm⁻^1^, amide N–H bending at 1541 cm⁻^1^ as well as N–H stretching at 3200–3400 cm⁻^1^, which belong to PNIPAM. In conclusion, FTIR spectra also supported the successful preparation of the block copolymer.

### 3.3. Self-Assembly Study of PSAMA-b-PNIPAM

Amphiphilic block copolymers can spontaneously form various higher order structures in aqueous solutions with a size range of 10–200 nm. Their shapes are typically discovered as spherical when the length of a hydrophilic segment outnumbers that of a hydrophobic one [52,53], whereas other structures including rods, vesicles and cylinders are also reported on block copolymers with a very long hydrophobic block [54,55,56]. For the designed amphiphilic block copolymer PSAMA-*b*-PNIPAM, fatty-acid-based hydrophobic block PSAMA is the core and the thermoresponsive hydrophilic block PNIPAM is the shell of the micelle. 

**Figure 4:**
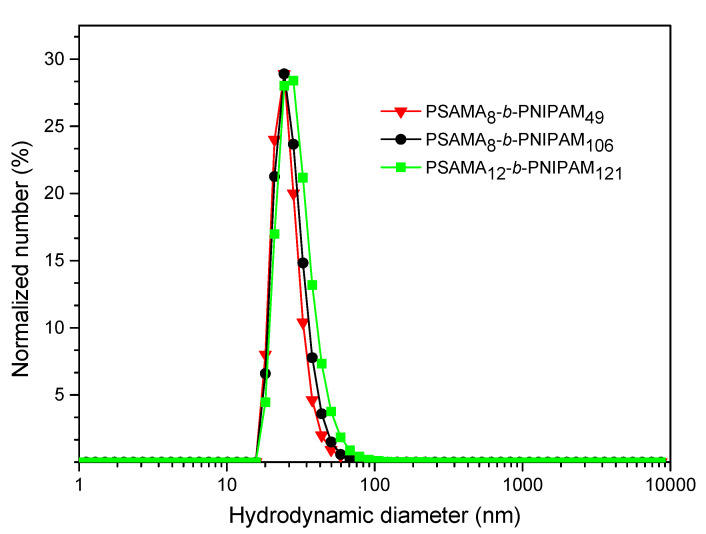
DLS distributions for PSAMA-*b*-PNIPAM in aqueous solution at 25 °C.

To study the self-assembly behavior of PSAMA-*b*-PNIPAM, the hydrodynamic diameter (D_h_) of block copolymer micelles was determined by DLS at 25 °C (Figure 4). Their average D_h_ values were measured as 27, 28 and 31 nm for PSAMA_8_-*b*-PNIPAM_49_, PSAMA_8_-*b*-PNIPAM_106_ and PSAMA_12_-*b*-PNIPAM_121_, respectively. The size of nano-assemblies was increased with increasing PSAMA block length and molecular weight of the block copolymer, which proves that micellar size can be well tuned by adjusting molecular characteristics of both blocks. In drug delivery through the bloodstream, the size and shape of nanocarriers play a crucial part in their biodistribution, circulation time and cellular uptake. A range of 10–100 nm diameter was reported to be the most ideal because this size carrier can efficiently avoid clearance by the kidney and recognition by the reticuloendothelial system (RES), resulting in longer circulation time [57,58]. More importantly, these carriers can selectively extravasate from the leaky vasculature around tumor sites based on EPR effect and accumulate at the target sites [59]. 

**Figure 5:**
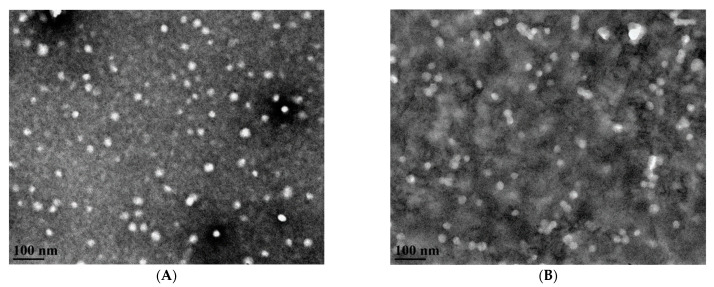

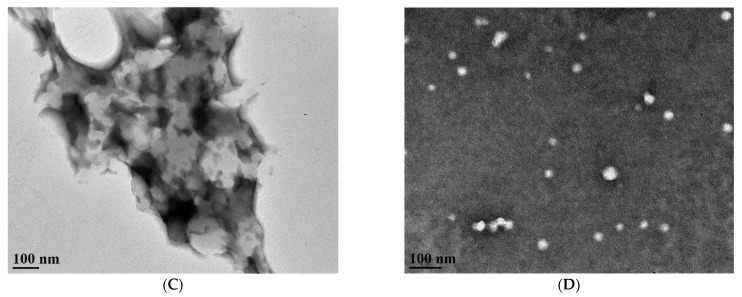
TEM images of (**A**) PSAMA_8_-*b*-PNIPAM_106_; (**B**) PSAMA_12_-*b*-PNIPAM_121_; (**C**) PSAMA_8_-*b*-PNIPAM_106_ micelle solution heated at 50 °C; (**D**) CBZ-loaded PSAMA_8_-*b*-PNIPAM_106_.

The morphology of self-assembled block copolymer particles was further explored by TEM. As displayed in Figure 5, all the PSAMA-*b*-PNIPAM copolymers with different block ratios self-assembled into spherical core-shell micelles. From TEM images, the micellar sizes were observed in the approximate range of 10–40 nm. It is noteworthy that unlike individual well-dispersed spheres formed by PSAMA_8_-*b*-PNIPAM_106_ (Figure 5A), the micelles prepared by PSAMA_12_-*b*-PNIPAM_121_ showed some aggregation behavior (Figure 5B). This may be as a result of longer hydrophobic content, the lipophilic tail of stearic acid being less mobile and incompactly packed during the micelle formation leading to their interactions with hydrophobic chains from other micelles. Moreover, it is interesting to note that when micelle solution was heated at 50 °C, PSAMA-*b*-PNIPAM micelles rapidly agglomerated and precipitated in aqueous solution (Figure 5C). This proved the thermoresponsive nature of synthesized micelles, suggesting that PNIPAM has transitioned from hydrophilic to hydrophobic.

### 3.4. Critical Micelle Concentration (CMC) Determination

Amphiphilic molecules begin to aggregate and self-assemble as their concentration reaches the threshold concentration called CMC. CMC is a significant indication of polymeric micelles’ stability in light of their application as targeted drug carriers [60]. Upon injection into the bloodstream, micelles are diluted by large volume of blood and those with high CMC value may disassemble into unimers, leading to premature release of encapsulated drugs. In the present study, CMC was determined by fluorescence technique using pyrene as the probe. Pyrene is a hydrophobic fluorescent probe with strong sensitivity to the surrounding microenvironment. During micelle formation, pyrene preferably transfers from polar solution to a hydrophobic core within micelles, causing a red shift in excitation spectrum maximum of pyrene from 333 to 337 nm. Hence, the ratio of fluorescence intensity at two wavelengths, 337 and 333 nm (I_337_/I_333_), were plotted against the logarithm of PSAMA-*b*-PNIPAM concentration for measuring CMC values. 

**Figure 6:**
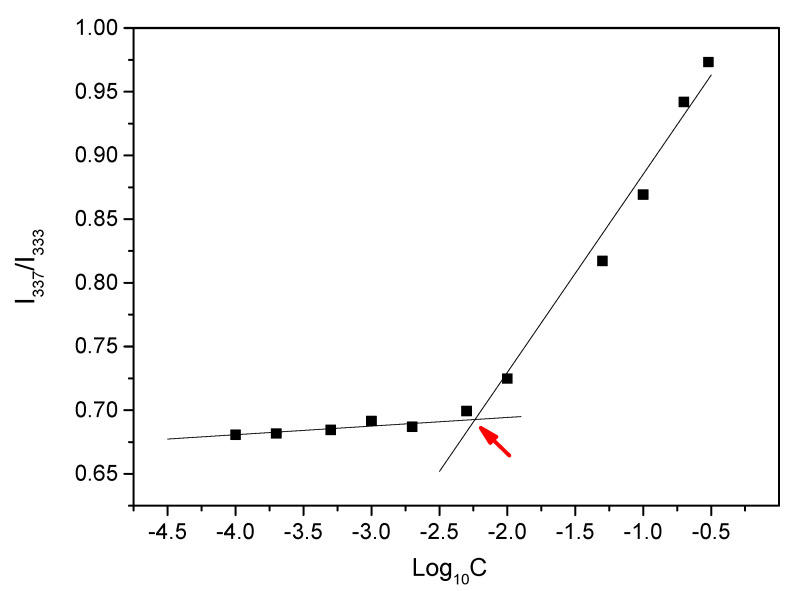
Plots of the intensity ratio I_337_/I_333_ of pyrene excitation spectra versus logarithm of concentration for PSAMA_12_-*b*-PNIPAM_121_.

As shown in Figure 6, below a certain concentration, I_337_/I_333_ values remained relatively steady. Above this concentration, there was a sharp increase of I_337_/I_333_ values, implying the incorporation of pyrene into the micelle hydrophobic core. The intersection point of the two straight lines through plots of I_337_/I_333_ ratios versus the block copolymer concentration was referred to as CMC. In this study, the CMC values of PSAMA_8_-b-PNIPAM_49_, PSAMA_8_-*b*-PNIPAM_106_ and PSAMA_12_-*b*-PNIPAM_121_ were measured as 0.0036, 0.0119 and 0.0058 mg/mL, respectively. Their CMC values decreased with the increasing PSAMA ratios in the block copolymer chains. The obvious dependence of CMC values on the length of the hydrophilic/hydrophobic segment can be explained as: increased hydrophobicity strengthens the interactions between hydrophobic chains, thus enhancing the aggregation tendency of amphiphiles to form micelles and thereby lowering the CMC. This trend is in accordance with previous reports [51,61]. 

### 3.5. Drug Loading and Release Behavior of Block Copolymer PSAMA-b-PNIPAM

Carbamazepine (CBZ), a lipophilic drug to treat epilepsy and nerve pain, was used as a model drug to investigate the drug loading efficiency and in vitro release performance of PSAMA-*b*-PNIPAM micelles. The CBZ-loaded polymeric micelle was also studied by TEM. TEM imaging (Figure 5D) showed that the drug-loaded PSAMA-*b*-PNIPAM micelles maintained their spherical morphology as expected but their size was approximately 42 nm, which was slightly larger than that of the blank micelles. To explore the impact of hydrophilic and hydrophobic chain length on drug loading efficiency, several PSAMA-*b*-PNIPAM were prepared with different block lengths and their DLE and DLC are listed in Table 2. Among these block copolymers, the maximum drug loading efficiency and drug loading content of 31.6% and 2.9% were observed for PSAMA_8_-*b*-PNIPAM_49_. The length and structure of core-forming segment play a significant role in drug loading efficiency as hydrophobic drugs are physically entrapped into a hydrophobic core via hydrophobic interaction. Therefore, a generally greater volume of core-forming polymer can contribute to higher encapsulation efficiency. However, in the case of this study, the reason that PSAMA_8_-*b*-PNIPAM_49_ exhibits the highest drug loading capacity may be associated with the balance between hydrophilic and hydrophobic blocks in aqueous medium. Combined with results observed by TEM for PSAMA_12_-*b*-PNIPAM_121_, the partial aggregation behavior of micelles may also impede the drug loading into the micellar core. It is especially important to note that the process conditions for CBZ encapsulation were not optimized to maximize DLE and DLC, but primarily to demonstrate the capability of these micelles as drug carriers. Factors affecting the drug-loading efficiency of polymeric micelles include polymer-drug ratio, molecular weight of the block copolymer, polymer-drug compatibility and encapsulation methods [62]. When employing the solvent evaporation method to prepare drug-loaded self-assemblies, type of organic solvent, rate of addition of solvent, organic solvent to water ratios and stirring rate all have a significant impact on the entrapment of drugs in nanoparticles. 

**Figure 7:**
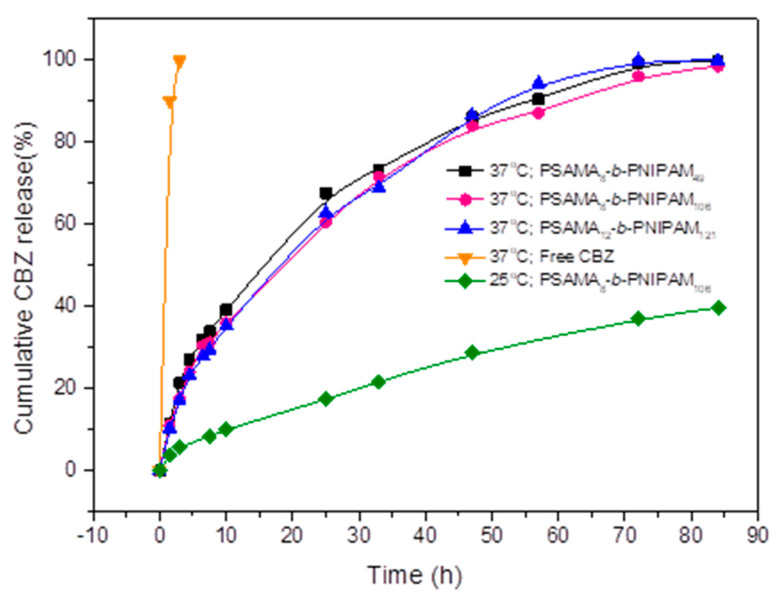
Release profiles in vitro of free CBZ and CBZ drug-loaded micelles in PBS (pH 7.4) at 37 and 25 °C.

The drug release behaviors of CBZ-loaded PSAMA-*b*-PNIPAM micelle formulations were investigated in simulated physiological conditions (0.01M PBS, pH 7.4). Different temperatures (25 and 37 °C) were applied to evaluate the thermo-sensitivity of PSAMA-*b*-PNIPAM micelles. As presented in Figure 7, the CBZ release amount and rate at 37 °C was far more dramatic than that at 25 °C, demonstrating an obvious temperature-triggered response. The percentages of CBZ released from PSAMA_8_-*b*-PNIPAM_106_ were about 20% and 65% within 36 h, and 35% and 92% within 72 h at 25 and 37 °C, respectively. The accelerated drug release at 37 °C is due to the phase transition behavior of PNIPAM above its LCST. This leads to collapse and shrinkage of block copolymer micelles, and consequently causes the drug to diffuse out more quickly. Relatively, the drug release rate at 25 °C may depend mainly on the micellar dissociation and polymer degradation.

The in vitro release of free CBZ was also conducted for comparison. The data showed that as for free CBZ release at 37 °C, approximate 90% CBZ was released into the medium in the first two hours and it took about three hours in total for its complete release. Whereas the release behavior of CBZ loaded in copolymer micelles was quite different under the same conditions. A typical two-phase release profile was found with a relatively rapid release phase during the initial period owing to the release of CBZ, which was located at the interface of the shell (PNIPAM segment) and the core (PSAMA segment), followed by a sustained and slow release up to 84 h at body temperature. No significant difference of release pattern was observed among the three drug-loaded PSAMA-*b*-PNIPAM micelles and they all showed good control for CBZ release at 37 °C.

## 4. Conclusions

In summary, we have synthesized the temperature-responsive amphiphilic block copolymer PSAMA-*b*-PNIPAM comprised of stearic-acid-based methacrylate polymer and PNIPAM by RAFT polymerization under microwave irradiations. Well-controlled block copolymers with variable block length were prepared by controlling the feeding ratio of monomers to macro-CTA. These amphiphilic block copolymers could spontaneously be assembled into spherical micelles with an average size range of ~30 nm. The balance between hydrophobic and hydrophilic segments had an impact on morphology, CMC and drug encapsulation performance of PSAMA-*b*-PNIPAM micelles. When increasing the block lengths of the stearic acid segment, the CMC values decreased due to the stronger hydrophobic interaction, whereas micelles showed bit aggregation attributing to the long hydrophobic tail of fatty acid and thus leading to lower encapsulation efficiency. Furthermore, 31.6% of CBZ can be loaded into PSAMA-*b*-PNIPAM micelles when the mass ratio of micelles to drug was fixed at 10:1. Improvement of drug-loading efficiency can be implemented by optimization of the self-assembly process and polymer to drug ratios in future studies. Complete release of drug from PSAMA-*b*-PNIPAM micelles with a sustained release behavior was observed at 37 °C. Overall, these results suggest that these block copolymers can be potentially used for controlled delivery of drugs. These findings indicate an opportunity to explore the utilization of renewable materials as replacements of non-renewable materials for smart delivery systems.

## Figures and Tables

**Table 1:** Microwave-assisted RAFT polymerization of PSAMA, PNIPAM and PSAMA-*b*-PNIPAM at 60 °C.

Polymer ^a^	[M]/[CTA]/[I]	Time (min)	Conv. ^b^	M_n_ ^c^ (g/mol)	M_w_/M_n_ ^c^	M_n,nmr_ ^b^ (g/mol)	Composition ^b^ SAMA: NIPAM
PSAMA_8_	25:1:0.2	20	28.6%	4730	1.08	3580	100:0
PSAMA_12_	50:1:0.2	20	23.5%	6820	1.10	5160	100:0
PSAMA_21_ *	25:1:0.2	300	69.3%	10,500	1.09	8610	100:0
PNIPAM_58_	150:1:0.2	30	35.6%	5640	1.06	6960	0:100
PSAMA_8_- *b*-PNIPAM_49_	100:1:0.2	25	75.6%	9320	1.10	9120	14:86
PSAMA_8_- *b*-PNIPAM_106_	300:1:0.2	25	62.3%	11,430	1.15	15,570	7:93
PSAMA_12_- *b*-PNIPAM_121_	300:1:0.2	25	64.1%	14,570	1.18	18,860	9:91

^a^ The subscripted numbers denote the degree of polymerization of each corresponding block. ^b^ Determined by ^1^H NMR. ^c^ Determined by GPC in THF. * Conventional heating method.

**Table 2:** Drug loading characteristics of CBZ-loaded micelles.

	DLE (%)	DLC (%)
PSAMA_8_-*b-*PNIPAM_49_	31.6	2.9
PSAMA_8_-*b-*PNIPAM_106_	24.4	2.2
PSAMA_12_-*b-*PNIPAM_121_	22.2	2.0

## Data Availability

The data presented in this study are available on request from the corresponding author.
